# How far will a behaviourally flexible invasive bird go to innovate?

**DOI:** 10.1098/rsos.160247

**Published:** 2016-06-15

**Authors:** Corina J. Logan

**Affiliations:** SAGE Center for the Study of the Mind, University of California, Santa Barbara, CA 93106, USA

**Keywords:** behavioural flexibility, innovativeness, string pulling, tool use, grackle, Icteridae

## Abstract

Behavioural flexibility is considered a key factor in the ability to adapt to changing environments. A traditional way of characterizing behavioural flexibility is to determine whether individuals invent solutions to novel problems, termed innovativeness. Great-tailed grackles are behaviourally flexible in that they can change their preferences when a task changes using existing behaviours; however, it is unknown how far they will go to invent solutions to novel problems. To begin to answer this question, I gave grackles two novel tests that a variety of other species can perform: stick tool use and string pulling. No grackle used a stick to access out-of-reach food, even after seeing a human demonstrate the solution. No grackle spontaneously pulled a vertically oriented string, but one did pull a horizontally oriented string twice. Additionally, a third novel test was previously conducted on these individuals and it was found that no grackle spontaneously dropped stones down a platform apparatus to release food, but six out of eight did become proficient after training. These results support the idea that behavioural flexibility is a multi-faceted trait because grackles are flexible, but not particularly innovative. This contradicts the idea that behavioural flexibility and innovativeness are interchangeable terms.

## 1. Background

While behavioural flexibility is considered a key factor in the ability to adapt to changing environments, it is difficult to quantify because it might be expressed in some contexts but not others [1,2]. A recent review characterizes behavioural flexibility as one's ability to change behaviour as circumstances change and inhibit behaviours that were previously successful [2]. Behavioural flexibility is one trait that can contribute to facilitating innovations, defined as inventing a new behaviour or using an existing behaviour in novel circumstances [2]. This is in contrast with most of the literature on behavioural flexibility and innovation, which seems to use these terms synonymously (e.g. [3–7]). There is a clear benefit to the revised framework because experimental evidence shows that these traits do not positively correlate: behavioural flexibility (reversal learning) and innovation (problem solving speed) are inversely correlated in Indian mynas and Florida scrub-jays [8,9] and there is no correlation in spotted bowerbirds, song sparrows, New Zealand robins and great-tailed grackles [10–13]. Because behavioural flexibility is multi-faceted, and because the links between behavioural flexibility, problem solving and innovativeness are rarely investigated from multiple angles in a given species (but see [8]), it is poorly understood how behaviour is used to react to changing circumstances.

Research on behavioural flexibility is accumulating on great-tailed grackles (*Quiscalus mexicanus*; family Icteridae; hereafter referred to as grackles) [11], a generalist forager that eats vertebrates, invertebrates, grains and fruits in open, grassy, water and urban areas [14–16]. Originating in Central America, they have become one of the most invasive species in North America [16,17]. Grackles are a likely candidate for having high levels of behavioural flexibility because invasion success is presumed to rely on behavioural flexibility [4], though their invasion success could also be due to an expansion of human modified environments, which is their preferred habitat [16]. For non-native species, invasion success is linked with introduction effort; however, exceptions exist [18]. Such exceptions could be due to species differences in traits that facilitate adapting to novel environments (e.g. range size, diet and habitat breadth), which have been found to positively correlate with invasion success [19]. At some point in their history, some aspects of human-modified environments to which grackles adapted were novel, such as fields of crops, dairies, garbage dumpsters, parking lots and outdoor cafes [15]. These novel aspects would probably have required a degree of flexibility to exploit in terms of habituating to humans or cows, learning what is edible and discovering new access methods. Some grackles are behaviourally flexible in that they can change their preferences when a task changes in two different contexts: a colour association task and in Aesop's Fable water tube tasks [11]. However, it is unknown how far they will go to invent new behaviours or use existing behaviours to solve novel problems. To investigate whether they invent new behaviours, I gave grackles two novel tests that a variety of other species can perform: stick tool use and string pulling.

A diverse range of bird species is reported to use tools to extract resources (36 species from eight orders use ‘true tools’ where objects are held in the beak or foot to form an extension of the body (e.g. probes, hammers, scoops) [20,21]). Several reports of stick or stick-like tool use are reported in Corvida and Passerida (but not specifically in grackles) [20,21]. Additionally, reports based on wild behaviour might not be indicative of what a species is capable of: rooks are not reported to use tools in the wild, but they can spontaneously make and use tools in captivity [17]. I predict that grackles will be able to innovate stick tool use because they are related to many species that perform such behaviours (they are in the order Passerida), they are generalist foragers [22] and they regularly turn over stones to look underneath for food [14, p. 322]. Keas are also generalist foragers, they overturn stones and they innovate tool use in the laboratory [23]. Therefore, this combination could indicate a propensity to access to food in more complicated ways. If grackles do not innovate stick tool use in this experiment, they will have the opportunity to socially learn to do so from a human demonstrator. Grackles probably attend to social information because they forage and roost in flocks [14], one individual foraging is likely to attract more individuals [24], and, when a foraging innovation was reported in California (eating dead insects from the licence plates of parked cars), many individuals were observed performing the behaviour, all using the same technique [25].

A wide range of species is known to innovate string pulling, where food is attached to the end of a piece of string which must be pulled in an iterative process to bring the food within reach. Some species spontaneously exhibited string pulling behaviour: 9 of 9 blue tits [26], 5 of 6 captive ravens and 1 in the wild [27,28], 6 of 25 goldfinches and 13 of 21 siskins [29], 1 of 12 great tits [30], 6 of 12 canaries (only juveniles were successful) and 4 of 18 greenfinches [31], 3 of 4 New Caledonian crows [32], 6 of 7 keas [33] and 2 bumblebees (other bumblebees socially learned to string pull) [34]. Some species were tested in a group setting, therefore it is unclear which individuals learned to pull strings by innovating and which through social learning: 19 of 19 rooks [35], 7 of 22 spectacled parrotlets, 8 of 10 rainbow lorikeets, 4 of 4 green-winged macaws and 3 of 3 sulphur-crested cockatoos [36]. Most birds build nests [37], as do bumblebees [38,39], which requires actions such as pulling and manipulating strands of nest material during construction [37]. I predict that grackles will innovate string pulling because of the diverse range of other species that have innovated this solution and because they also build nests [15], which might have given them the necessary experience to transfer these skills to a new situation.

## 2. Material and methods

### 2.1. Subjects

Eight adult great-tailed grackles (50% female) were caught in the wild using a walk-in baited trap (0.61 m high × 0.61 m wide × 1.22 m long; design from [29]). Birds were caught in Santa Barbara, California, USA at the Andree Clark Bird Refuge in September 2014 and released in December 2014 (four birds: Tequila, Margarita, Cerveza, Michelada; batch 1) and at East Beach Park in January 2015 and released in March 2015 (four birds: Refresco, Horchata, Batido, Jugo; batch 2). See [11] for full details.

### 2.2. Experimental set-up

Grackles were housed individually in aviaries (183 cm high × 119 cm wide × 236 cm long) at the University of California, Santa Barbara, USA. Individual housing should not be more stressful for this species than social housing because they are polygamous with weak social bonds and do not exhibit many affiliative behaviours (only proximity is described in their ethogram) [15,40]. Testing was conducted at the front of each aviary in visual isolation, while at the back of the aviary neighbours could often see each other because birds were allowed to peck holes in the tarpaulins dividing the aviaries. Grackles had ad libitum access to water at all times and unrestricted amounts of food (Mazuri® Small Bird Food composed of pressed granules) were accessible for a minimum of 20 h d^−1^. On testing days, their main diet was removed for up to 4 h while they participated in experiments and could eat bread or peanuts if successful. Test apparatuses were placed on tables (60 cm wide × 39 cm long) and rolled into each aviary by the experimenter who then left the aviary for testing sessions, which lasted up to approximately 20 min. Any disturbance that was caused by the experimenter entering the aviary immediately subsided after the experimenter stepped out of the aviary, as indicated by the bird's landing on the table to participate in the task. Grackles were already habituated to humans because of their urban foraging habits, which include stealing food from tables at outdoor cafes (C.J.L. 2012, personal observation). Their first 5 days in the aviary were spent habituating them to a human presence just outside their aviary door where experimenters stood when conducting the experiments. All grackles adjusted well to this set-up as indicated by their willingness to interact with the apparatuses in these and other experiments (see [11]).

#### 2.2.1. Experiment 1: stick tool use

**Figure 1.**
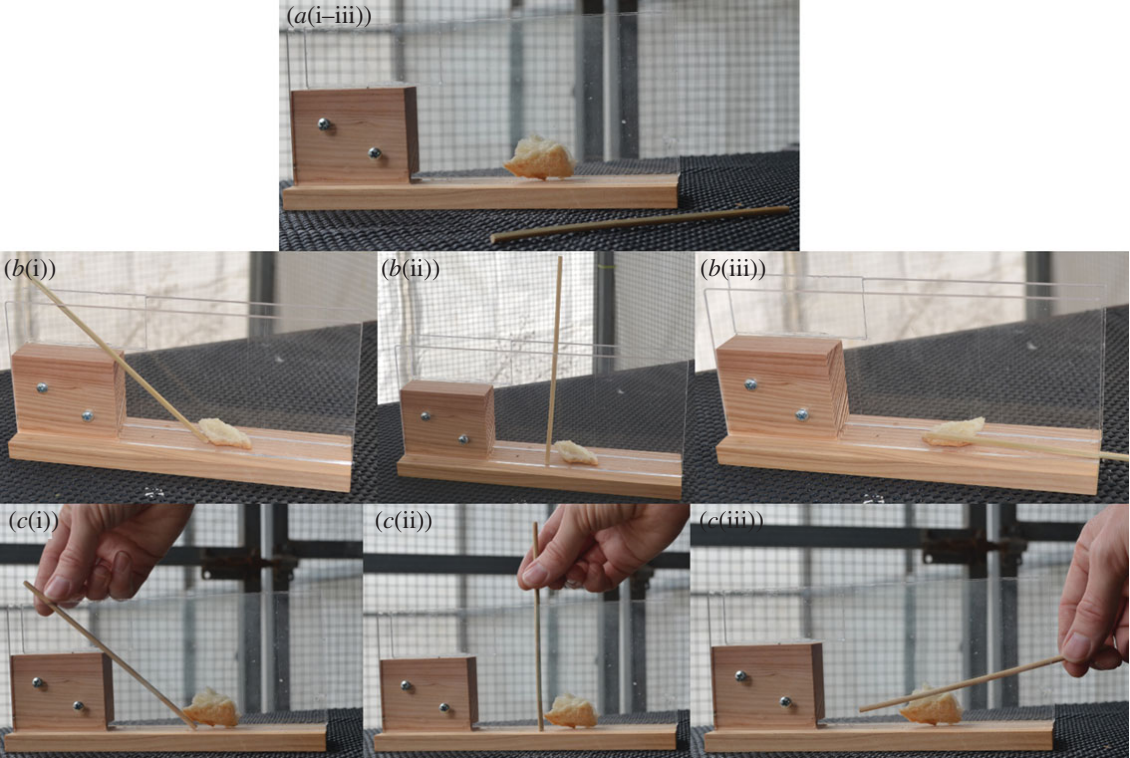
To determine whether grackles innovate stick tool use, three experiments were conducted. In Experiment 1.1, the stick was placed on the table in all three 5 min trials (*a*(i–iii)). In Experiments 1.2 (*b*(i–iii)) and 1.3 (*c*(i–iii)), the stick was inserted into the apparatus for three consecutive trials, each with a different position: position 1 (left; trials 1, 4, 7, 10, 13), 2 (middle; trials 2, 5, 8, 11, 14) and 3 (right; trials 3, 6, 9, 12, 15). Experiment 1.2 had three 5 min trials and Experiment 1.3 had three 5 min trials per day for 5 days totalling 15 trials where each trial began with a demonstration from the experimenter.

Grackles were presented with a stick and an apparatus containing an out-of-reach piece of food to determine whether they would innovate stick tool use (the apparatus was similar to the crevice platform described in [41]). Trial durations, conditions and passing criterion followed methods that were previously used for Goffin cockatoos [42] to make grackle results comparable. The apparatus had clear acrylic walls and a wooden floor (figure 1). One end had a wooden barrier while the other end and the top were open (9.1 cm tall × 19.3 cm long × 2.0 cm wide). Birds were habituated to the apparatus by placing it on the table and baiting it with a reachable peanut piece near the entrance. If they were unwilling to approach the box and eat the food, the box was left in their aviary overnight and they were fed their meals from it until they habituated. If a bird readily ate the reachable peanut during a habituation trial, they immediately began Experiment 1.1. During experimental trials, the box was placed on the table with a large piece of out-of-reach bread and a bamboo stick weighing 2 g for 5 min per trial in three consecutive trials [42]. A bird was considered successful if they used the stick to obtain the bread. If a bird was successful or if it grabbed the stick and tried to use it on the bread, 15 more trials were conducted to determine whether they performed the action by chance or were consistently able to solve the task.

*Experiment 1.1: pick up and use a stick.* I placed the stick on the table next to the box (figure 1*a*(i–iii)). If a bird was not successful or did not try to use the stick on the bread in the three trials, which were given one immediately after the other, then it moved on to Experiment 1.2.

*Experiment 1.2: use an inserted stick.* I inserted the stick into the box so that one end touched the bread while the other protruded enough for the bird to grab. The stick position changed for each of the three trials that were conducted one immediately after the other. In trial 1, the stick protruded from the rear of the box (figure 1*b*(i)), in trial 2 the stick was oriented vertically (figure 1*b*(ii)) and in trial 3 the stick protruded from the front (figure 1*b*(iii)). If a bird was successful or if it grabbed the stick and tried to use it on the bread, then 15 more trials were conducted. If a bird was not successful or did not try to use the stick on the bread in the three trials, then it moved on to Experiment 1.3.

*Experiment 1.3: use an inserted stick after a human demonstrates the solution.* The methods were the same as Experiment 1.2, except that I demonstrated how to use the tool to obtain the bread three times at the beginning of each trial in each of the three respective stick positions (figure 1*c*(i–iii)). Three 5 min trials (one for each stick position) were conducted, one immediately after the other, per day for 5 days for a total of fifteen 5 min trials.

#### 2.2.2. Experiment 2: string pulling

I followed methods that were previously used for New Caledonian crows (horizontal string pulling, 42; vertical string pulling, 31) to make grackle results comparable. Grackles were habituated to two different types of string (described below) by tying one piece between two perches in their aviary (as in [28]).

**Figure 2.**
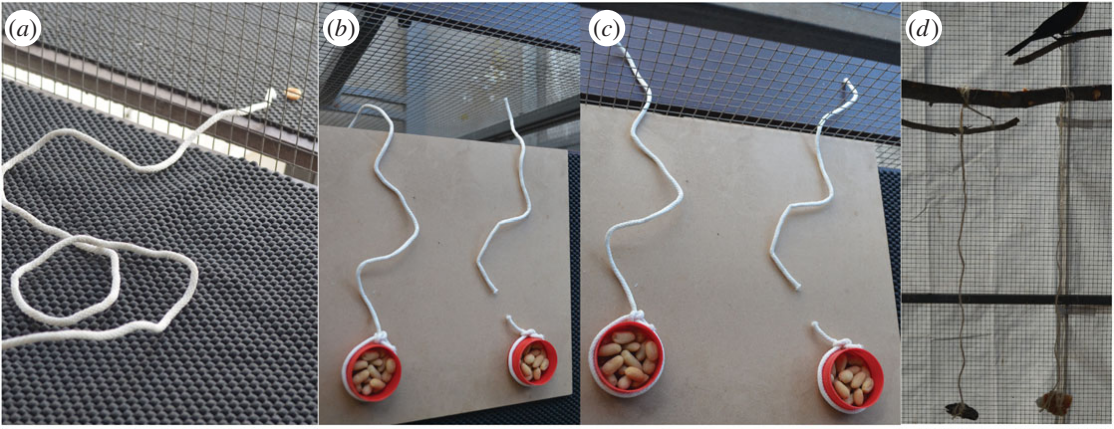
To determine whether grackles innovate string pulling behaviour, two string pulling experiments were conducted: horizontal (*a*–*c*) and vertical (*d*). A habituation trial with a string inserted into the aviary next to a peanut (*a*), the observation period of the horizontal string pulling experiment with a choice between a broken string and a string that is connected to a cup of peanuts (*b*), the interaction period of the horizontal string pulling experiment where the bird had the opportunity to choose and pull a string (*c*) and the vertical string pulling experiment where they had the choice between pulling up a string with a block of wood or a piece of bread hanging at the end (*d*).

*Experiment 2.1: horizontal string pulling: broken versus connected.* Two snaked strings (white shoelaces with the plastic ends cut off) were each attached to a cup containing 17 peanuts (cup + peanuts + string = 20–21 g; figure 2*b*). One string was connected to the cup and one string was broken with a 3 cm gap near the cup (similar to [31]). The strings and cups sat on a table outside the aviary with the string ends sticking through the wire mesh wall onto a table inside the aviary so the bird could land and interact with the string. Birds were habituated to the snaked string by placing the string (without the food cups) on a table outside their aviary with one end sticking inside their aviary next to a peanut piece on a table so they could land and eat the peanut (figure 2*a*; grackles that passed habituation had two to six habituation sessions over the course of 2–5 days. Jugo did not pass habituation, but he was only given one habituation session because testing time ran out and he needed to be released back to the wild). If a bird did not eat the peanut after a few minutes, the tables were left in place and they were fed their dinner from the table inside their aviary while the string (now out of reach) was left on the table outside their aviary until they passed the habituation trial. If a bird readily ate the peanut in a habituation trial (within 1 min), they began Experiment 2.1.

In experimental trials, the bird was first presented with the apparatus out of reach for 20 s so they could observe the options and then the string ends were inserted into their aviary so they could interact with the string. Trials lasted 3 min after the string was made accessible and ended after the bird stopped interacting with their first string choice or until the time elapsed if they did not interact with the strings. If a bird did not come to the table within 3 min, a break was given (lasting from 5 min to 3 days) and the next trial was preceded by a habituation session. Habituation to strings and food cups was restarted if it appeared that the bird was avoiding the table because of a neophobic reaction (e.g. it refused to land on the table). If a bird did not interact with the string for three consecutive 3 min trials, which could occur one immediately after the other or on consecutive days, their experiment concluded. Four of the eight birds experienced Experiment 2.1 (horizontal string pulling) first (Tequila, Margarita, Cerveza and Michelada) and the other four birds experienced Experiment 2.2 (vertical string pulling) first.

*Experiment 2.2: vertical string pulling: wood versus bread*. Two strings (twisted sisal twine) were tied 15 cm apart on a perch (1.35–1.47 m above the ground), each attached to an object suspended 50 cm below the perch (figure 2*c*; perch circumference: 4.9–8.8 cm; similar to [20,21]). From one string hung a 10 g piece of wood (bark) and from the other hung an 8–10 g piece of dried bread (weights included string). Objects were similarly shaped, but the volume of the bread was larger than the wood, which resulted from making them similar weights. To ensure that the lack of an interaction was not due to neophobia, birds were required to pass a habituation trial before starting the experiment. Habituation trials, where strings with objects attached as in the experimental set-up were present, lasted 20–60 min with multiple placements of peanuts on the perch equidistant between the two strings (one to two sessions over the course of 1–2 days for grackles that passed habituation; grackles that did not pass habituation were given three to eight sessions over the course of 1–9 days). Birds were also habituated to bread and wood wrapped in string by placing them in their food dish (as in [28]). Once a bird readily ate the bait, they moved on to the experimental trials. Experimental trials began when the bird touched the hanging part of the string and lasted until it left the perch or pulled up and made contact with the object or food reward for a total of 20 trials [32]. If a bird did not interact with the hanging part of the string, up to three 20 min trials were conducted to allow enough time for them to attempt the task and then their experiment concluded.

## 3. Results

Watch video clips showing examples of the experiments at: https://youtu.be/VqcyrIGk8Bc.

### 3.1. Experiment 1: tool use

No grackle used the stick on the out-of-reach bread in any experiment (1.1, 1.2 or 1.3), therefore all birds were unsuccessful (table 1). Six out of eight grackles (Margarita, Tequila, Horchata, Refresco, Batido and Jugo) interacted with the stick by pulling it out of the box and setting it on the table, manipulating it inside the apparatus (but without the stick touching the food), picking it up and carrying it around and putting the stick back in the apparatus, eventually losing interest in the stick after the interaction. Two grackles (Michelada and Cerveza) did not interact with the stick at all. Batido, Jugo, Refresco and Horchata picked up the stick starting in trial 1 in Experiment 1.1, and, starting in Experiment 1.2, they, as well as Margarita (starting in trial 1), pulled the stick out of the apparatus. Tequila pulled the stick out of the apparatus (starting in trial 3) only after seeing demonstrations in Experiment 1.3. Additionally, Batido manipulated the stick inside the apparatus (starting in trial 3 of Experiment 1.2); however the stick did not act on the bread, therefore it did not count as a success. Refresco (starting in trial 7) and Horchata (starting in trial 10) also did this after the demonstrator trials started (Experiment 1.3).

**Table 1.** Results for each bird in each experiment: all individuals failed to innovate tool use (did not use the stick on the bread) and string pulling and their level of interaction with the stick or string was noted (X, did not pass habituation). Y, yellow; P, purple; B, blue; O, orange; R, red; G, green.

bird (colour rings)	sex	tool use	string pulling: vertical	string pulling: horizontal
Tequila (YP)	M	interacted stick	did not touch hanging string	pulled string
Margarita (PB)	F	interacted stick	X	no interaction
Cerveza (BO)	F	no interaction	did not touch hanging string	no interaction
Michelada (OR)	F	no interaction	X	no interaction
Batido (OP)	M	interacted stick	X	no interaction
Horchata (GR)	F	interacted stick	did not touch hanging string	X
Refresco (PY)	M	interacted stick	X	no interaction
Jugo (RB)	M	interacted stick	X	X

### 3.2. Experiment 2: string pulling

For the grackles that experienced horizontal string pulling before vertical string pulling, most showed an interest in the horizontal string pulling experiment: Margarita and Cerveza came to the table, but Michelada did not. However, Tequila was the only grackle to touch the strings. In Tequila's first trial, he briefly touched the connected string first; however, this was not counted as a choice due to experimenter error and then he pulled the broken string all the way onto his table. In his third trial, he pulled the connected string, bringing the peanut cup closer to him (but not within reach), then he stopped interacting with the string and his trial ended when he approached the other string. After his third trial, Tequila stopped interacting with the strings (trials 4, 5 and 6) and his experiment ended. No grackles solved the subsequent vertical string pulling task: they readily ate the bait placed on the branch between the two strings; however, they only pulled at the little strands of string that stuck out where the string was attached to the branch (thus not causing the food to move) and not the hanging part of the string that was attached to the food.

For the grackles that experienced vertical string pulling before horizontal string pulling, only one, Horchata, participated in the vertical string pulling experiment and she was unsuccessful in the same way as the grackles above. Batido, Refresco and Jugo did not pass habituation: they did not eat the bait on the branch between the two strings during habituation trials. None of these grackles passed the subsequent horizontal string pulling experiment: they came to the table, but did not interact with the string. The approach of the onset of the breeding season potentially distracted the males (Batido, Refresco and Jugo), which might have reduced their interest in participating in new experiments.

## 4. Discussion

Contrary to the prediction, no grackle innovated stick tool use even after observing a human experimenter demonstrate the solution many times. Their performance is similar to Goffin cockatoos where most individuals also did not innovate stick tool use and some also did not learn by observing a conspecific demonstrator [42]. The reasons why such individual and species differences exist remain speculative. Though grackles are generalist foragers, perhaps they attend to particular features of their environment that differ from generalist foragers in other species where one or more individuals have innovated tool use (New Caledonian crows [43], keas [23] and Goffin cockatoos [42]). Most grackles showed an interest in the apparatus and the stick, indicating that they were motivated to participate and some persisted for extended periods of time in attempting to access the bread. Their failure to persist more in their interactions with the stick could come from a lack of dexterity for manipulating such objects in their bill. Those that pulled the stick out or manipulated it in the apparatus did so awkwardly. It is hypothesized that New Caledonian crow eye positions and bill shape have co-evolved with their tool using abilities, resulting in an increase in binocular vision and a straighter bill, which make it easier to manipulate tools [44]. Compared with New Caledonian crows, grackles' eyes are set more to the sides of their heads and both mandibles are curved, which could impede their ability to manipulate sticks. Indeed, such bill morphology has been hypothesized to reduce keas ability to use stick tools [23]. In the absence of reinforcement, which could allow an association to form between the correct part of the task (the stick) and the food, the incentive to persist with the stick could be easily extinguished.

It is possible that grackles attend more to conspecific than to heterospecific behaviour and would have learned to innovate tool use from a conspecific demonstrator. However, this seems unlikely because tool use demonstrations by a heterospecific appeared to influence grackle behaviour throughout the course of Experiment 1.3. Tequila interacted with the stick on multiple occasions, but only after seeing the solution demonstrated. He pulled the stick, which indicates that he was probably drawn to interact with the stick because the demonstrator touched it (stimulus enhancement), rather than imitating the particular movements (push and pull rather than just pull). Also, Refresco and Horchata changed how they interacted with the stick: rather than just pulling the stick out of the box, they began to move it around inside the box after seeing demonstrations, which might indicate they copied actions. An experiment designed to test what social and asocial learning mechanisms grackles use would provide insights into whether and how information spreads through groups.

Also contrary to the prediction, grackles did not innovate vertical string pulling and did not successfully innovate horizontal string pulling. Grackles performed similarly to New Caledonian crows on the horizontal string pulling task in that only one bird in each species actually pulled the connected string enough to move the food [45]. Though in the grackle's case this positive reinforcement was not enough to continue pulling to reach the reward and he extinguished string pulling behaviour shortly thereafter. Grackles failed to interact at all with the hanging string in the vertical string pulling experiment, which contrasts with results from other bird species in which some to all individuals spontaneously pulled hanging strings (see Introduction for details). Grackles underwent similar habituation protocols to ravens and both species showed neophobic reactions to hanging strings with objects attached [28]. The ravens eventually overcame their neophobia because all individuals solved the task [28], whereas grackles were perhaps less motivated to overcome their neophobia [46]. Those grackles that were not neophobic and participated in the experiment engaged with the task (interacted with the strings), but not correct part of the task (the hanging part of the strings), indicating they might not have persisted enough in their attempts to solve the task [46].

A third novel test of innovative behaviour was previously conducted on these particular grackles, which involved dropping stones down a platform apparatus to release a food reward [11]. Several corvid (birds in the crow family) species either learn to drop stones very quickly (e.g. in 1–33 trials for rooks, Eurasian jays and some New Caledonian crows) or over the course of several testing sessions (e.g. up to 255 trials for western scrub jays and some New Caledonian crows) [47–52]. Only two birds so far have spontaneously dropped stones without training: a New Caledonian crow [53] and a rook [43], though the rook could have socially learned how to solve the task. The presumption is that the more rapidly an individual learns, the more innovative it is and the less it relies on operant conditioning to form a positive association between the action and the successful receipt of the reward. Thus, corvids vary in their innovativeness, not only across species, but also within species. Grackle performance was similar to slow-learning New Caledonian crows [51] and western scrub jays [52]: no grackles innovated stone dropping and the ones that became proficient learned to drop stones in 100+ trials, indicating they relied on operant conditioning rather than innovation to solve the task proficiently. Long periods of learning to drop stones down tubes probably did not confound the subsequent tests of behavioural flexibility because the flexibility tests were conducted in a different context (water-filled tubes rather than a platform apparatus) and grackles were presented with novel choices, where one choice was more functional than the other [11]. Their prior learning about stone dropping did not inform them about which choice to make during the flexibility experiments where flexibility was determined by an individual's choice of novel objects in changing circumstances.

Two of these three novel tests involved tool use and grackles are not known to use tools. What can an investigation of a non-tool user in a tool-using context tell us about innovation and tool use more broadly? Both tool using and non-tool using species did not innovate stone dropping behaviour, indicating that species with repertoires including tool use are not necessarily at an advantage in terms of being able to more easily generalize their skills [7]. Stick tool use is a behaviour that occurs in several bird species and a few mammal species [21] and in species not known to use tools in the wild (rooks [47], keas [23], Goffin cockatoos [54]). Perhaps, the main factor that determines whether a species invents tool use in the wild regards costs and benefits [55]. For grackles, food is abundant year-round in their human-modified environment, thus reducing the need to invent tool use. However, this does not explain why grackles did not invent tool use in an experimental setting, whereas a few other bird species could. One reason a natural history-based explanation might remain elusive is publication bias. It is unclear which other species do not innovate stick tool use because studies showing an absence of such behaviour are less likely to be published [56,57], which could also result in the reduction of attempts to explore this behaviour at a broad taxonomic scale. If present, such publication bias would obscure potential explanations and impede the development of future hypotheses. Further investigations are needed to determine whether food abundance in the wild is related to a lack of innovating tool use in the laboratory.

In general, juveniles are known to be more neophilic than adults in that they are more willing to approach novel objects in the absence of a food reward [58]. Perhaps, juvenile grackles would have expressed more interest in the settings that evoked neophobic reactions in these adults, which might have resulted in more persistent behaviour, potentially leading to the correct solution. Future research is required to test this hypothesis, though I predict that juveniles and adults will not differ in neophilia because this species is closely linked with human environments and they routinely interact with novel objects, which probably require exploration to determine whether food is present.

## 5. Conclusion

These results support the idea that behavioural flexibility is a multi-faceted trait because grackles exhibit behavioural flexibility (they adapt responses to changing circumstances [11]), but are not particularly inventive when it comes to creating new behaviours for novel problems. This contradicts the idea that behavioural flexibility and innovativeness are interchangeable terms, which is often assumed (e.g. [3,4]), and supports the revised framework where behavioural flexibility is an independent trait that can be one of many mechanisms facilitating innovation [2]. Thus, a lack of innovativeness should not be considered evidence of a lack of behavioural flexibility.

Grackles have a wide variety of foraging behaviours (e.g. they hunt tadpoles and fish by wading in shallow water, fish by flying close to the water's surface, overturn objects to seek food underneath, remove parasites from cattle and extract larvae and insects from grassy and recently plowed areas) [14], therefore perhaps rather than inventing new behaviours to solve novel problems, they instead apply their existing behaviours to new contexts. Indeed, so far they performed well in experiments that rely on their existing behaviours such as searching for hidden food [11]; however, further experiments are needed to test this hypothesis. These results support the idea that each facet of behavioural flexibility must be investigated independently [1,2] to make sound hypotheses about how species actually, rather than hypothetically, adapt their behaviour to changing environments.

## Data accessibility

Data from Experiment 2 are reported here and data from Experiment 1 are available at the KNB Data Repository [59].
